# Adult Stem Cell Responses to Nanostimuli

**DOI:** 10.3390/jfb6030598

**Published:** 2015-07-16

**Authors:** Penelope M. Tsimbouri

**Affiliations:** Centre for Cell Engineering, Institute of Molecular, Cell and Systems Biology, College of Medical, Veterinary and Life Sciences, Joseph Black Building, University of Glasgow, Glasgow G12 8QQ, UK; E-Mail: penelope.tsimbouri@glasgow.ac.uk; Tel.: +44-141-330-7555; Fax: +44-141-330-7663

**Keywords:** stem cells, stem cell niche, MSC, nanoforces, nanotopography, nanovibration, mechanotransduction, ECM, cell adhesion

## Abstract

Adult or mesenchymal stem cells (MSCs) have been found in different tissues in the body, residing in stem cell microenvironments called “stem cell niches”. They play different roles but their main activity is to maintain tissue homeostasis and repair throughout the lifetime of an organism. Their ability to differentiate into different cell types makes them an ideal tool to study tissue development and to use them in cell-based therapies. This differentiation process is subject to both internal and external forces at the nanoscale level and this response of stem cells to nanostimuli is the focus of this review.

## 1. Introduction

Adult stem cells, occasionally referred to as somatic stem cells, are undifferentiated cells, found among differentiated cell populations within a tissue. They can self-renew and can differentiate (multipotent) to produce the essential specific cell types of the tissue they reside in (stem cell niche). The primary role of adult stem cells is tissue homeostasis. In contrast to the embryonic stem cells (not the focus of this review) that originate from the pre-implantation-stage embryo, the origin of adult stem cells in some mature tissues is still to be elucidated. Stem cells in their niche respond to different biophysical factors operating at the nanoscale, which play a major role in stem cell fate. These factors can be chemical or mechanical, such as matrix elasticity, the local nanotopography, nanovibration and nanoforces that can be internal or external to the cell. Work supporting these conclusions is reviewed.

### 1.1. Adult Stem Cells

Discovery and subsequent research on adult stem cells has generated great enthusiasm. Adult stem cells have been discovered in more different tissue types than it was once believed possible. This has led scientists and clinicians to investigate the possible use of adult stem cells in transplantation.

In the 1950s, advances in stem cell research led researchers to discover two types of stem cells in the bone marrow. The first population, called hematopoietic stem cells (HSCs), gives rise to all the types of blood cells in the body and have been used in transplants for more than 40 years [1]. The second population, the bone marrow stromal stem cells, also called mesenchymal stem cells (MSCs) or skeletal stem cells (SSCs) by some, were discovered a few years later. MSCs make up a small proportion of the stromal cell population in the bone marrow, and research has shown that *in vitro*, with the addition of growth factors, they can differentiate into bone, cartilage, fat, cells that support the formation of blood, and fibrous connective tissue [2,3,4]. However, in recent years, research indicated that using just nanotopography, scientists can induce differentiation without the need for growth factor supplements [5,6]. The need for growth factors is a major issue when culturing cells with the potential of direct transplantation into patients as this could trigger immune response and rejection of the transplanted cells or the cells fail to differentiate. The third population, endothelial stem cells (ESCs) are multipotent and like other stem cells they can self-renew and differentiate. These stem cells give rise to progenitor cells, which are intermediate stem cells that lose their potency and develop into endothelial cells (ECs). ECs create the thin-walled endothelium that lines the inner surface of blood vessels and lymphatic vessels [7].

In the 1960s, researchers identified dividing cells with the potential to become nerve cells in two regions of the brain. Despite this, it was only in the 1990s that it was generally accepted that the adult brain contains stem cells with the potential to generate the three major cell types in the brain—the two non-neuronal cell types, astrocytes and oligodendrocytes, and nerve cells or neurons [8].

Adult stem cells and their corresponding niches were identified in many more tissues including muscle [9], mammary gland [10], testis [11], liver [12], intestine [13,14], heart [14], white fat [15,16] and skin [17].

### 1.2. The Stem Cell Niche

The concept of a stem cell niche was first reported by Schofield *et al.* in 1978. In the niche, stem cells reside, interacting with other cells types and help to control tissue homeostasis [18]. A germ-line stem cell niche from *Drosphila melanogaster*, was the first stem cell niche identified and was first reported in 2000 [19]. It is widely accepted that stem cell niches exist in most, if not all, tissues, and that they provide cells with mechanical support, growth factors, optimum physical and chemical conditions, as well as stem cell-specific self-renewal and differentiation cues (reviewed in [20]). Scientists have identified stem cell niches associated with different stem cell types in mammals such as haematopoietic, neural, skin and intestinal [21,22,23,24,25,26,27,28,29].

Whilst a definitive stem cell niche associated with the different stem cell types have not yet been identified, there have been many proposals for certain locations within the relevant tissues. For instance, the crypts of the small intestine, are the considered the hub of the self-renewal process of the intestinal epithelium [30,31,32,33,34,35].

The skin is a very complex tissue and under normal conditions the epidermis, sebaceous glands, and hair follicles are thought to be maintained by their own dedicated adult stem cell populations residing in three distinct microenvironments: the basal layer of the interfollicular epidermis (IFE), the follicular bulge, and the base of the sebaceous gland [36]. The bulge area functions as a niche, where epithelial stem cells [37] are situated and maintained [38,39]. Epithelial stem cells are multipotent and when tissue homeostasis is disrupted, (*i.e*., in the presence of wounds) they reveal their plastic potential by contributing to the regeneration of all three structures. They self-renew and travel to either the IFE to serve as epidermal progenitors for generating epidermal cells or they migrate at the base of the sebaceous gland to convert to hair-matrix progenitors, that will further give rise to the hair shaft [37,40,41].

Mammary gland stem cells (MGSCs) are quiescent and able to self-renew like other stem cells. They reside in the mammary gland and they can differentiate into ductal, alveolar and myoepithelial cells [10]. MGSCs are well studied due to their contribution to development and adaptive changes in response to different hormonal stimuli as well as their involvement in breast tumorigenesis [42].

Adult neurogenesis takes place in two main regions of the brain: the subventricular zone (SVZ) and the subgranular zone (SGZ). It has been suggested that in both regions, astrocytes, glial cells that have long been considered as just support cells in the brain, are neural stem cells and the areas they reside is the neural stem cell niche [23,43,44,45,46].

Haematopoietic stem cells (HSCs), as mentioned earlier, are multipotent, self-renewing progenitors that generate all mature blood cells. HSC function is tightly controlled to maintain haematopoietic homeostasis, and this regulation relies on specialized cells and factors that constitute the haematopoietic “niche”, or microenvironment [22,24,47].

The exact niche for MSCs has not yet been identified. It has been proposed that MSCs reside in the bone cavity, within the endosteal [48,49] and perivascular niches [50,51]. The perivascular location for MSCs is suggestive of a crossover with pericytes [51]. Furthermore, MSCs can be derived from other tissues (e.g., fat, umbilical cord and dental tissue) and thus have other niches. However as MSCs have also been extracted from nonvascularised niche tissue (e.g., cartilage) this may indicate that different MSC populations exist.

The MSC/HSCs niche is perivascular and usually, located near trabecular bone. Endothelial cells, sharing a common lineage with haematopoietic cells, are thought to make up the cellular element of the niche and synthesise multiple factors that promote HSC maintenance and localization [52].

It is possible to identify key factors that appear to be essential for maintaining the niche environment by using previously identified stem cell niches. Regulation of self-renewal or differentiation depends on different factors such as (a) external physical interactions of stem cells with other cell types in the vicinity, the basement membrane and extracellular matrix (ECM) (b) intrinsic and extrinsic signalling from other cells within and out with the niche, as well as (c) neural and metabolic signalling [53,54].

Adult stem cells are often assumed to be quiescent within their niche, dividing infrequently to generate one stem cell copy and a rapidly cycling cell. The rapidly cycling cells (transit-amplifying cells) then undergo a limited number of cell divisions before terminally differentiating into the functional cells of that tissue.

## 2. Adult Stem Cells React to Nanoenvironment

**Figure 1:**
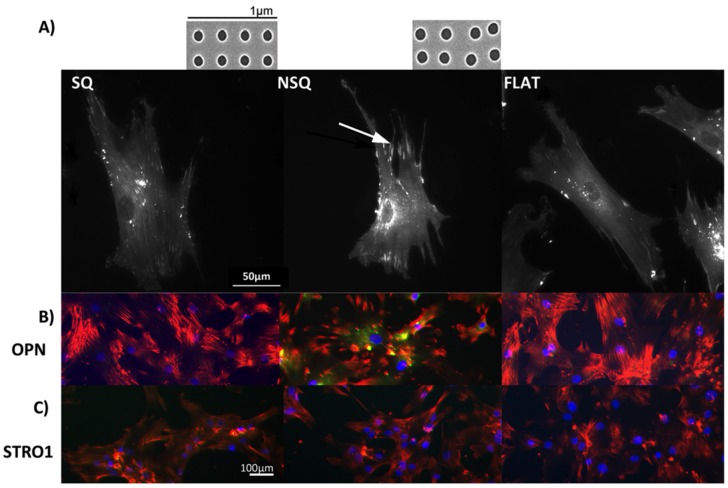
Fluorescent microscopy images of mesenchymal stem cells (MSCs). (**A**) MSC morphology and attachment on nanopit substrates. (i) On highly ordered nanotopography (shown in inset above SQ, SEM of square (SQ) 120 nm diameter pits, 100 nm deep, average 300 nm centre-centre spacing in a square arrangement), cells appeared less-spread and stellate in shape displaying small adhesions; (ii) On a disordered nanotopography (shown in inset above NSQ, SEM near square (NSQ), as before but with up to ±50 nm placement error), MSCs were spread with large lamellae and adopted an elongated cell shape. Super-mature adhesions (arrow) were observed in these cells; (iii) The MSCs morphology/adhesion pattern on the flat control surface appeared to be of an intermediate state of the two test topographies; (**B**) Fluorescent microscopy of MSC differentiation on nanotopographies. The MSCs cultured on NSQ showed strong expression of the bone marker osteopontin (OPN). Red = actin, green = OPN, blue = nucleus; (**C**) Fluorescent microscopy images of cell surface markers for MSC self-renewal. Only MSCs cultured for 4 weeks on the SQ surface express the MSC marker STRO-1. Red = actin, green = STRO-1 blue = nucleus. MSCs on planar controls did not express either of these genes after the 4 weeks culture.

Tissues provide their resident cells with a topographically complex environment consisting of neighbouring cells and other tissue connective intercellular materials such as collagen. It has been reported that cells can react to such “active” nanotopography by changes in adhesion and also in gene expression, *i.e.*, Kruppel-like Factor 2 (KLF2) and endothelin 1 in Le-2 strain cells (mouse lung endothelial cells expressing CD34) [55,56,57,58,59]. Changes in the transcriptional machinery affect gene and protein expression and also cell behavior. Studies on MSCs have revealed combination effects of nanotopography and nanovibration on gene expression (see below). Such effects are more common on cells grown on stationary nanotopography, after the cells have been subjected to shear flow forces, and can last up to several hours of treatment. One effective nanotopography, the near-square 50 (NSQ50) caused a substantial increase cell adhesion and changes in gene expression in comparison to two other related patterns (patterns shown and described in Figure 1). However, very few patterns have been tested to help us fully understand the phenomenon [60,61].

### 2.1. Reactions to Nanoforces

When cultured on nanotopographical surfaces, cells experience changes in the form of self-generated forces that act on the cells due to changes in their adhesion points [62,63]. To further study this, Curtis and colleagues examined the effects of applying nanoscale mechanical forces to the cells [56,57,64,65,66]. Application of nanoscale forces (of 1 pN resulting in 5–15 nm displacement per cell) leads to runt related transcription factor 2 (RUNX-2) expression in MSC cells [65,66]. These nanoforces are similar to the mechanical forces applied on the ear hair cells involved in hearing but smaller than the blood flow-caused shear forces in the capillary endothelium. Wu *et al.* reported an effect on electrical responses of osteocyte-like cells to hydrodynamic pressure waves exerting forces of 1 – 2.3 pN in localised parts of the cell in the form of charge transfer across the cell membrane of the order of 1 nC over a period of less than 2 s [67].

### 2.2. Reactions to Vibration

Cells respond to either external or internal vibration forces. Pre *et al.* showed that stimulation at frequencies around 30 Hz induces adipose-derived stem cells to differentiate into bone [68]. Similarly, Kim *et al.* [69] reported that daily exposure to vibrations increased the proliferation of hMSCs, with the highest efficiency occurring at vibrations at 30 to 40 Hz. Specifically, these conditions in 2D cultures promoted osteoblast differentiation through an increase in alkaline phosphatase activity and *in vitro* matrix mineralization. In 3D cultures however, hMSCs showed increased expression of type I collagen, osteoprotegrin, or VEGF, and VEGF induction [69]. Nikukar *et al.* applied vibrations to MSCs at frequencies between 1 and 50 Hz and established the role of nanovibrations in gene expression. The group further stimulated the cells with higher frequencies and obtained additional changes in osteogenic (*i.e*., Runx2, osteocalcin) gene expression [65,66]. This could either be the effect of stimulation acting on resonant structures inside the cell, or that energy inputs into the cell rise as frequency increases generating impulses of similar intensity per unit time.

Temperature fluctuations caused by such stimulation could contribute to the effects observed; however, in the study by Nikukar *et al.*, lower energy inputs caused temperature rises of less than 1 °C [65]. Nevertheless, based on the literature, temperature measurements have not been considered in research involving greater movements and often fairly high frequencies. There is more extensive literature on the effects of larger scale vibrations [70,71,72].

Nanovibrations have further been investigated for hMSC differentiation into different tissues types. Work using human vocal fold fibroblasts (hVFF) and bone marrow mesenchymal stem cells (BM-MSC) stimulated at higher frequencies at 200 Hz suggested that BM-MSC may be a suitable alternative to hVFF for vocal fold tissue engineering [73].

## 3. Biomaterials Highlight Stem Cell Responses to Nanostimuli

The *in vitro* use of biomaterials is an essential tool to assess the role of mechanical cues *in vivo.* In many of the studies discussed in this review, MSCs have been the stem cells of choice partly due to the fact that they are easily accessible but also due to their multipotential to differentiate into different cell types such as osteoblasts, adipocytes [2], chondrocytes [74], neural marker expressing cells [75], myoblasts [6,76], fibroblasts, and stromal cells [77]. MSC self-renewal however, is still under investigation.

Biomaterials have been used over the years to study the effect of changes of the physical environment on cells, chemistry and topography (see review by [78]). The rationale for developing nanostructured materials for clinical applications originates from the complicated physicochemical structure of extracellular tissue *in vivo*. Studies have indicated that most cells react significantly to nanotopographical cues *in vivo* [79,80].

### 3.1. Elasticity

MSCs and differentiated cells have their own unique physical properties such as stiffness (Table 1). However, the cells within the tissues are embedded inside a very complex fibrous extracellular matrix (ECM). The physical and mechanical properties of the ECM are essential for tissue homeostasis, through regulating cellular functions such as attachment, spreading, migration, stem cell differentiation and proliferation [81,82]. The ECM has been implicated in the pathogenesis of cancer [83,84,85].

**Table 1:** Young’s Modulus Measurements of hMSC using different techniques.

Cell	Young’s Modulus	Measure Technique	Publication
hMSC	Instantaneous: 0.5 kPa Equilibrium: 0.1 kPa 3.2 kPa Spread: 3.2 kPa Spherical: 2.5 kPa	Micropipette aspiration AFM indentation AFM indentation	[86,87,88]
Adipocytes	Spread and Spherical 0.61 kPa	AFM indentation	[88]
Adipogenice differentiation	Instantaneous: 0.42 kPa Equilibrium: 0.09 kPa	Micropipette aspiration after 21 days	[86]
Neural cells	Pyramidal neurons: elastic modulus between 480 Pa at 30 Hz and 970 Pa at 200 Hz. *E’* of astrocyte somata was between 300 Pa at 30 Hz and 520 Pa at 200 Hz.	Scanning force microscopy	[89]
Chondrocyte	Spread and Spherical: 1.2 kPa Instantaneous modulus:1.06 ± 0.82 kPa Relaxed modulus of 0.78 ± 0.58 kPa Apparent viscosity: 4.08 ± 7.20 kPa	AFM Indentation Unconfined creep cytocompression and digital video capture	[88,90]
Osteoblast	1.75 kPa Spread: 5.8 kPa Spherical: 2.0 kPa	AFM indentation AFM indentation	[87,88]
Osteogenic differentiation	Instantaneous: 0.9 kPa Equilibrium: 0.2 kPa	Micropipette aspiration after 21 days AFM indentation after 10 days	[86,87]

Engler *et al.* studied the effects of matrix elasticity on stem cell phenotype [6,91]. They showed that a stiff matrix of 34 kPa supported osteogenic differentiation, a medium elasticity matrix of 11 kPa induced myogenic differentiation and a soft matrix of 0.1 kPa supported differentiation of MSCs into neuronal-like cells.

Gilbert *et al.* [92] studied the importance of the elastic modulus of the cell microenvironment on the muscle stem cell (MuSC) self-renewal and muscle homeostasis. Using an *in vivo* mouse model, they found that when MuSCs are cultured on medium elasticity matrix (12 kPa), they can self-renew and can potentially be used to restore damaged muscle tissue when transplanted *in vivo* [92].

Recent work on the effects of matrix elasticity on MSCs differentiation, has identified two major players of mechanotransduction triggered by ECM rigidity and cell shape, YAP (Yes-associated protein) and TAZ (transcriptional coactivator with PDZ-binding motif, or WWTR1), both closely regulated by the Rho GTPase activity and the actomyosin contractility resulting from cell adhesion to the ECM [93,94]. Yang *et al.*, cultured MSC on hydrogels with different stiffness and showed that YAP/TAZ act as an intracellular mechanical stiffness sensor providing MSCs with mechanical memory [95].

### 3.2. Chemistry

Recent advances using chemistry to produce patterned surfaces for culturing cells has provided us with the ability to define the composition of a surface in a precise manner. Specific types or density of ligands has helped us to understand the role of individual ECM components on stem cell adhesion and differentiation. Moreover, single-cell shape studies can be performed without the interference of cell density or neighboring cells. Chemical surfaces are usually produced by microcontact printing (µCP) using self-assembled monolayers (SAMS) and, SAMS presenting a maleimide group for peptide immobilization [96,97,98,99].

#### 3.2.1. µCP

McBeath *et al.* used µCP to change MSC cell density and hence cell spreading and demonstrated the effect of mechanical stimuli in MSC differentiation and lineage commitment [100]. In addition, they identified a key role of RhoA in mechanotransduction. Further work by Killian *et al.* using µCP to change cell shape, showed the ability to alter lineage commitment of MSCs cultured on star-shaped patterns with sharp edges (osteogenic) and flower-shaped patterns with soft edges (adipogenic) as a result of changes in acto-myosin contractility on the pattern shapes [101]. This work suggests that changes in cell shape can lead to changes in both cell contractility as well as the cell’s responsiveness to changes in ECM.

Connelly *et al.* also using µCP were able to control epidermal stem cell differentiation by changing different parameters, *i.e.*, cell shape, ECM density and/or composition. However, in contrast to the previous studies on MSCs, this study showed that the levels of G-actin, dependent on cell spreading on the µCP surfaces, controlled the activity of serum response factor (SRF), a key mediator of terminal differentiation [102,103].

#### 3.2.2. SAMs

SAMs have enabled scientists to mimic the ECM composition and density in a very precise manner. Using SAMs, Cavalcanti-Adam *et al.* generated different nanopatterns using the arginine-glycine-aspartic acid (RGD) motif and identified the fundamental mechanism of the membrane protein integrin binding and focal adhesion (FA) formation [104]. It was suggested that the combination and availability of the proteins involved in the focal adhesion formation created a minimum lateral distance requirement over which binding can occur.

The density and affinity of RGD ligands on a surface can affect MSC differentiation. Scientists used different densities of high and low affinity RGD ligands and found that they could control MSC differentiation down to the osteo-, myo- and neurogenic lineages in a ligand density- and affinity-dependent manner [105].

The highlighted studies illustrate the importance of mechanical cues on MSC differentiation and introduce the concept of how mechanical cues affect gene expression and hence MSC differentiation.

#### 3.2.3. Other Chemistries

With the exception of the defined protein ligands, studies on surfaces with different chemical composition and functionality have revealed some interesting facts. Curran *et al.* reported that simple surface chemistry such as OH, CH_3_, COOH or NH_2_, attracts the appropriate serum proteins in culture, controlling MSC differentiation [106]. Using precise patterning of CH_3_-modified surfaces Curran *et al.* further showed that this methodology retains MSC surface markers and their self-renewal [107].

The chemical functionality for MSC differentiation potential has been the target for a few studies including the development of array-based methods to screen large libraries of different chemistries [108,109]. Furthermore, 3D hydrogel scaffolds carrying phosphate and *t*-butyl functionalities were able to induce controlled MSC differentiation down to the adipogenic and osteogenic lineages, respectively [110].

## 4. Nanotopographical Effects on Stem Cells Fate

ECM consists of a complex of proteins and nanoscale features to which cells respond. Over the last 20 years, research has shown that the nanotopographic characteristics of the substrate on which cells reside play a major role in cell adhesion. In this early work the nanofeatures were e-beam fabricated pillars or pits arranged at various arrangements (e.g., ordered squares, hexagonal or random geometries) [57,111,112,113,114,115].

Cell-topographical interactions, using different cells types, have been shown to affect different cellular functions such as adhesion, morphology, gene expression and proliferation, [115,116,117,118,119,120]. Stem cell research indicated that changes in gene expression affect stem cell fate [5,75]. More recent work on nanotopographical effects on stem cells fate, showed major changes on focal adhesion size and orientation which in turn resulted in cytoskeletal changes, altering cell shape, chromosome territory shifts and hence, gene expression [61,121]. Tsimbouri *et al.* [61] using nanoscale sized pits with highly ordered and slightly disordered geometries, showed that MSCs developed longer focal adhesions (Figure 1a, arrow) with up-regulated expression of osteogenic differentiation markers (e.g., osteopontin, OPN) on the slightly disordered nanopits (Figure 1b). In contrast, MSCs on the ordered nanopits, had a smaller focal adhesion size, they self-renewed and continued to grow as multipotent stem cells as indicated by expression of MSC markers (e.g., STRO1) (Figure 1c). The focal adhesion length changes were shown to have a direct effect on intracellular tension, with the osteogenesis requiring higher levels of tension, self-renewal an intermediate level and adipogenic require a lower tension level [60,61,122].

Osteogenic differentiation of MSCs has been also observed on surfaces such as TiO_2_ nanotubes [123]. Yim *et al.* cultured MSCs on nanogrooved surfaces and these cells differentiated to express neural markers [75]. Phosphorylated FAK was found to be a major player in signal transduction regulating cell fate through focal adhesions.

MSC have been the most preferred stem cell type to study response to topography, however, other stem cells such as neural and embryonic have also been investigated [124,125,126,127,128,129].

## 5. Mechanotransduction Signalling

### 5.1. Cell: Extracellular Matrix Adhesions (ECM)

ECM is a complex fibrous and protein rich structure. In general, whilst the structures of individual ECM components are diverse, many share common structural motifs such as the RGD motif found on the hydrophillic loops of a number of ECM molecules including fibronectin, vitronectin and tenascin. RGD is the most common motif, and is essential for fibronectin and integrin mediated cell attachment [130].

**Figure 2:**
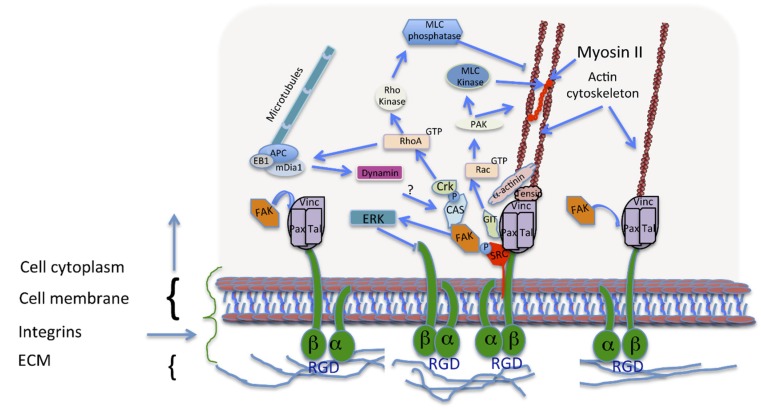
Focal adhesion (FA) formation and maturation. Immature adhesions, formed after integrin binding (right), are connected to the actin cytoskeleton via a protein complex involving linking proteins such as α-actinin, vinculin and talin. Signalling adaptors, FAK and paxillin, are recruited to these focal complexes (middle). FA formation, activates Rac, thus promoting actin polymerization and inhibiting myosin II coupling in the lamellipodium. These processes are required for the assembly and disassembly of different adhesions upon cell movement. During adhesion maturation the focal complexes develop into larger and longer FAs with the recruitment of more proteins like tensin (middle). Talin, vinculin, and p130Cas, have tension-sensitive conformations. RhoA activation is required for FA formation and actin bundling due to increased myosin II activity. Upon FA disassembly, dynamin is involved in the internalization of the integrins, and microtubule targeting.

ECM-cell interaction is mediated through transmembrane proteins called integrins [131]. Integrins bind to proteins in the ECM through complex mechanism (Figure 2) involving the formation of attachment points between the cell membrane and a surface resulting in tension formation within the cell and at the same time transmitting information from the ECM inside the cell. This transmission of information is mediated through the binding of a number of integrin-binding molecules, as RhoA kinase and focal adhesion kinase (FAK). These kinases can regulate multiple cellular processes such as proliferation and differentiation through the activation of a series of intracellular signaling pathways including extracellular signal-regulated kinase/mitogen-activated protein kinase (ERK/MAPK) [132,133].

As shown in Figure 2, the initial integrin-ECM protein contact leads in changes in their conformation and affinity, which in turn results in integrin clustering and immature focal complex formation. Subsequent recruitment of linker proteins, *i.e.*, vinculin, talin and paxillin, causes actin stress fiber formation. This process results in changes in cytoskeletal tension and the cell responds to this tension by changing the focal adhesion size [134].

The size of the integrin-mediated adhesions vary greatly and are divided into 3 groups: (a) focal complexes of approximately 1 µm in length (b) focal adhesions of 2–5 µm in length or (c) fibrillar adhesions 5–10 µm in length [120,135,136,137]. The fibrillar adhesions are sometimes called super-mature adhesions and their role in osteogenesis has been suggested [112]. Literature shows that these structures are found at the leading edge of cell motility structures called lamellipodia, where fast remodeling of adhesions takes place during movement, around the cell periphery as well as in the central areas of cells [138,139].

**Figure 3:**
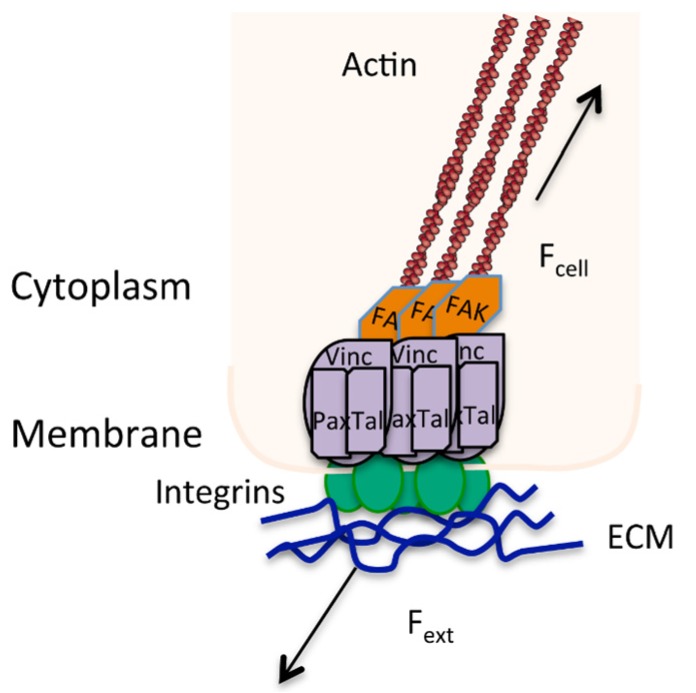
Focal adhesion showing the bi-directional signaling caused by a balance of external (*F*_ext_) and internal forces (*F*_cell_) in directing stress at a mechanosensor point. Actin stress fibres (brown) are anchored into focal adhesion complexes (*i.e*., vinculin, talin, paxilin, FAK) that are bound to the ECM through integrins (green).

Integrin binding allows bi-directional signals (Figure 3) to be relayed at the cell-material interface, hence allowing mechanical signaling from the ECM inside the cell or intracellular signaling causing ECM remodeling. There is a large volume of published material on the relation of focal adhesion size and intracellular tension in regulating MSC processes such as differentiation and self-renewal [101,119,121,140,141,142].

### 5.2. Integrin Mediated Mechanotransduction

**Figure 4:**
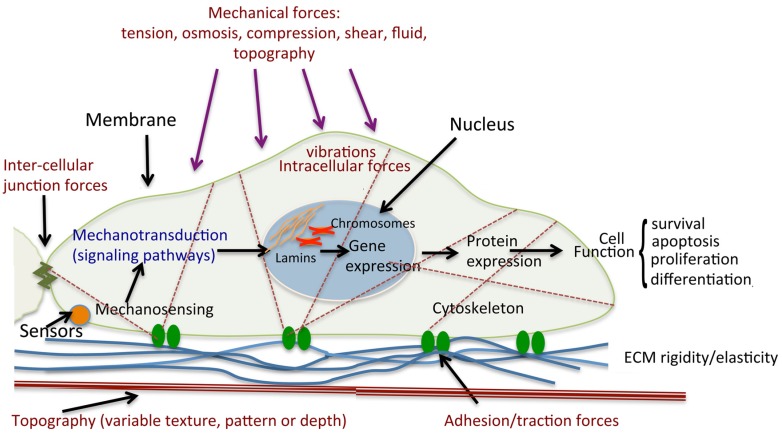
Schematic diagram of cell mechanical stimulation. Mechanical forces stimulate cells through the activation of mechanosensors, including the receptors that respond to ligands. Cells are exposed to different types of forces: extracellular such as shear forces through fluid flow over the cell surface, tensile/traction forces through the ECM, intercellular through contact with neighbouring cells, and intracellular cytoskeletally generated contractile forces (actomyosin contraction, microtubule polymerisation and depolymerisation, osmotic forces). Sensor activation leads to initiation of signaling cascades, and hence changes in gene expression. Such mechanotransduction results in modulations of protein expression and cellular functions such as survival, proliferation and differentiation. Illustrated is a single cell attached to a complex ECM through the focal adhesions.

The interaction between integrins and the ECM enable cells to transport information from the cell membrane to the nucleus and hence transform a mechanical signal into a biochemical signal, a process called mechanotransduction (Figure 4). The cell cytoskeleton is a complex structure that provides a structural support for the cell shape and movement but acts as the mechanical and biochemical link to the extracellular environment. The cytoskeleton consists of microfilaments, microtubules and intermediate filaments, which are responsible for cellular tensegrity [143,144]. Tensegrity is a structural principle that can be applied in architectural systems when opposing forces act together to maintain the shape of the structure, as well as providing strength and flexibility to the structure. In the cellular structure, cells are maintained in a prestressed state and are in equilibrium under a balance of intra- and extra-cellular forces. Any mechanical stresses applied to the cytoskeleton, via cell membrane proteins, are immediately sensed and cause the cell to react as a whole, resulting in a complete change in the cytoskeletal structure [145].

The complexity of the cytoskeleton provides the cytoplasm with a plasticity required for the transmission of the mechanical signals to the nucleus. The LINC complexes (linkers of nucleoskeleton and cytoskeleton) are the important mediators linking the cytoskeleton to the nucleoskeletal proteins, the lamins [146,147]. Research has shown that direct mechanotransduction is the result cytoskeletal changes that can directly affect lamin bound intermediate filaments at the nucleus. This binding changes the spatial arrangement of lamin-bound chromatin, chromosome packing or cause chromosome territory shifting affecting gene expression [145,147,148,149,150,151,152,153].

### 5.3. Integrins and the Cell Cycle

Adhesion and mechanical cues are directly involved in the regulation of G1 phase of the cell cycle. Earlier research has shown that cell spreading and intracellular tension are essential in controlling cell proliferation [154,155]. Subsequent research by Kornberg *et al.* showed that upon integrin binding, FAK is phosphorylated in a cellular spreading-dependant manner, with higher tension favoring increased FAK expression [156]. Phosphorylated FAK controls cell proliferation though activating the ERK2 kinase pathway, and induces cyclin D1, regulator of the G1/S cell cycle transition [156].

The composition of the ECM plays a key role in cell cycle control as different integrins have different effects. It was found that specific integrin subunits such as α5 and α 6promote cell cycle progression whereas integrin α2β1 has been shown to reduce proliferation in different cell types tested [157,158].

### 5.4. Integrins and Stem Cell Division

Integrin activation and crosslinking to other adhesion molecules are important factors in the regulation of cell polarity, mitotic spindle orientation and cell division [159,160].

Stem cells undergo symmetrical or asymmetrical division, which depends on the plane of cell division. For example, Thery *et al.* showed that the spatial distribution of the ECM and hence integrin arrangement are essential in directing the plane of cell division [161]. Similarly, Toyoshima *et al.* also used micropatterning in combination to integrin inhibition to dictate the orientation of the mitotic spindle and they found that individual integrins may regulate stem cell division [162]. In a different study with neural stem cells, inhibition of integrin binding leads to a modification in the plane of cell division and, as a consequence, a change from asymmetrical to symmetrical cell division [163].

Recent studies have shown that the orientation of cell division is important for dictating cell fate within the stem cell niche [164,165,166,167]. In the case of stem cells, symmetrical or asymmetrical outcomes can be regulated by mechanical cues that dictate the plane of cell division [168]. *In vivo*, the effect of chemical factors on daughter cells may be responsible for this for example Habib *et al.* showed that spatially restricted exposure to factors like wnt3a, a secreted signalling protein, may lead to asymmetric division of stem cells [169].

## 6. Mechanotransduction and the Primary Cilium

The primary cilia are small sensory structures and work as key coordinators of mechanical and chemical signals from the extracellular environment and transmit these signals to the nucleus to elicit a cellular response. They are microtubule-based organelles that emanate from the cell surface of most mammalian cell types during growth arrest into the extracellular space. They have been shown to function as mechanosensors, and signalling hubs for key signaling pathways such as hedgehog, non-canonical wnt, PDGF and calcium signalling [170,171,172,173,174,175]. Ciliogenesis is tightly regulated by the cell cycle, occurring during G0/G1, and cilia brake down at the onset of late G1/S phase [176].

In MSC’s and other stem cells, primary cilia have been shown to play a role in both mechanotransduction and chemically induced differentiation [170,177,178].

## 7. Future Perspectives and Clinical Implications

Over the past few decades, different therapeutic approaches using biomaterials have been developed and applied in regenerative medicine for the repair of different tissues [179,180,181]. Increased longevity of the human population and many other factors leading to loss or loss of function of organs and tissues due to accidents, disease or birth defects has led to a dramatic increase in the clinical demand to promote the regeneration of injured/diseased tissues.

Stem cell physiology and behavior are becoming well-understood and their use in regenerative therapy is very promising. *In vivo*, appropriate differentiation, proliferation and maintenance of potency are regulated by stem cells and their niche, their specific microenvironments [182,183]. Furthermore, molecular structures such as paxilin and talin inside the cell membrane and factors in the ECM (e.g., surface chemistry and geometry of nanofeatures) are crucial in stem cell responses to topography. Hence, biomaterials can be fine-tuned to mimic the stem cell niche and/or ECM structural complexity and specifically effect the *in vitro* differentiation, essential for clinical applications [184,185].

Using a variety of biomaterials in the absence of growth factors, our understanding of stem cells processes is greatly improved. For example, the use of, nanoscale topography, surface chemistry and tunable stiffness has aided our understanding of MSC adhesion, proliferation and differentiation requirements. These approaches can also be used on other stem cell types, aiding our understanding of different stem cell behaviour and hence complement the continuous search for stem cell regenerative therapies [186,187,188].

Nanovibrations have been shown to influence stem cell differentiation towards bone or vocal fold tissue cell type [56,66,68,69,73].

Vibration stimulus is widely known to be beneficial especially in the muscle tissue with therapeutic potential in cases of muscle disfunction due to disease, age or weight problems [189,190,191]. Whole body vibrations (WBV) is also used by some professional athletes to stimulate and strengthen damaged muscles. However, cell response to nanovibrations is a complicated process. Different *in vitro* studies have shown that hMSC mechanoreceptors may initially translate mechanical signals through the cytoskeleton to the nuclear compartment inducing differentiation down a particular lineage but this response is time dependent. Therefore, an extensive analysis of various nanovibration stimuli conditions such as different frequencies and time points to identify the optimal conditions for lineage specific differentiation is essential.

Hence, despite the extensive work to design and improve biomimetic materials for regenerative medicine, only a few biofunctionalized biomaterials have been successfully introduced into the clinic. The big hurdle is the fact that the tissue microenvironment is very complex and reproducing the *in vivo* conditions required for stem cell differentiation is very difficult.

Therefore, culture systems, materials and conditions required to accommodate and control the different levels of the tissue healing process need to be enriched with the appropriate physical (*i.e*., nanotopography, nanovibration) and/or chemical cues that would work in concert for stem cell differentiation and to promote tissue healing.

The design of such sophisticated nano- or micro-devices is highly attractive and with vast therapeutic potential.
